# Anti-Obesity Effects of SPY Fermented with *Lactobacillus rhamnosus* BST-L.601 via Suppression of Adipogenesis and Lipogenesis in High-Fat Diet-Induced Obese Mice

**DOI:** 10.3390/foods12112202

**Published:** 2023-05-30

**Authors:** Taewook Kang, Jin Ree, Joo-Woong Park, Hyewon Choe, Yong Il Park

**Affiliations:** 1 Department of Biotechnology, Graduate School, The Catholic University of Korea, Bucheon 14662, Republic of Korea; twkang@biostream.co.kr (T.K.); jree@biostream.co.kr (J.R.); 2 Biostream Co., Ltd., Suwon 10442, Republic of Korea; pjwrnds@biostream.co.kr (J.-W.P.); hwchoe@biostream.co.kr (H.C.); 3 Graduate School of Genetics and Biotechnology, Kyung Hee University, Yongin 17104, Republic of Korea

**Keywords:** *Lactobacillus rhamnosus*, probiotics, prebiotics, fermented sweet potato, anti-obesity

## Abstract

In this research, the potential anti-obesity efficacy of *Lactobacillus rhamnosus* BST-L.601 and its fermented product (named SPY) with mashed sweet potato paste were investigated using 3T3-L1 preadipocytes and high-fat diet (HD)-induced obese mice. SPY (0–0.5 mg/mL) dose-dependently and significantly reduced lipid accumulation and TG content and the expression of adipogenic markers (C/EBPα, PPAR-γ, and aP2) and fatty acid synthetic pathway proteins (ACC and FAS) in 3T3-L1 adipocytes, demonstrating that SPY suppresses adipocyte differentiation and lipogenesis. Oral administration of SPY (4 × 10^7^ CFU/kg body weight) to HD-induced obese mice for 12 weeks significantly reduced the body and liver weight, the size of adipocytes, and the weight of epididymal, visceral, and subcutaneous fat tissues. SPY was more effective in decreasing body weight gain in HD mice than in treatment with BST-L.601 alone. Administration of SPY or BST-L.601 also reduced the serum level of total cholesterol and LDL cholesterol and leptin secretion at a similar level. These results revealed that both SPY and BST-L.601 effectively suppress HD-induced adipogenesis and lipogenesis, suggesting that these materials would be useful in the functional foods industry to ameliorate and/or prevent obesity.

## 1. Introduction

Obesity, one of the major metabolic diseases, is being magnified as a worldwide health problem related to various fatal diseases such as cardiac dysfunction, diabetes, hypertension, osteoarthritis, and cancer [1]. Recently, the incidence rate of obesity has rapidly increased, as reported by the OECD in 2017. Moreover, a serious problem with this situation is childhood obesity, with an estimated 15.5% of OECD infants being obese [2]. Obesity is defined as an abnormal accumulation of body fat, resulting in excessive expansion and growth of adipose tissue due to an imbalance between energy intake and expenditure [3]. The development of obesity is defined by an increased adipose tissue mass that can be driven by either an unusually larger number or expanded fat cells (adipocytes) [4]. The expanded size of adipocytes (hypertrophy) is predominantly attributed to the accumulation of lipids (lipogenesis), and the increased cell number of adipocytes (hyperplasia) leads to the proliferation and differentiation of adipocyte precursor cells to mature adipocytes, which is a cellular process called adipogenesis [4]. Therefore, the mass of adipose tissue can be controlled by inhibiting adipogenesis, reducing the accumulation of lipids, improving lipolysis, and/or guiding the apoptotic death of adipose cells [5]. During the differentiation of adipocytes, several adipogenic transcription factors such as sterol regulatory element-binding protein-1c (SREBP-1c), peroxisome proliferator-activator receptor-γ (PPAR-γ), and CCAAT/enhancer binding protein-α (C/EBPα), are essential regulators of adipogenesis. The expression of C/EBPα, PPAR-γ, and several lipogenic enzymes, including fatty acid synthase (FAS) and acetyl-CoA carboxylase (ACC), is stimulated by SREBP-1c products [4,6]. Activation of lipogenic enzymes converts acetyl-CoA to fatty acids and triglycerides and induces tissue uptake into plasma [7].

To solve the obesity issue, studies on how to change the intestinal microbiome composition with probiotics such as Lactobacillus have been conducted in recent decades [8]. These results suggested that when the composition of the intestinal microbiome is changed, intestinal microorganisms (mainly lactic acid bacteria, LAB) influence energy consumption and lipid accumulation; thus, microorganisms of human intestines are useful for obesity control [9]. It was also reported that the composition of the intestinal microbial community was adjusted through oral administration of probiotics to induce obesity suppression, and the ingestion of LAB caused a change in the human intestinal microbial community, especially Lactobacillus species, for a long period of time, which could be different from obese people [10]. Probiotic strains, especially Lactobacillus genera, have proven anti-obesity effects by reducing fat mass and body weight [11]. The anti-obesity efficacy of probiotics can be demonstrated by increasing satiety and lowering insulin resistance [12]. As it has been recently suggested that intestinal microbes could cause obesity, the anti-obesity effect of controlling intestinal microbes through probiotics is being studied [13]. These mechanisms include modifying the composition of the gut microflora, improving barrier integrity in the intestine, producing beneficial metabolites, and regulating the host immune system [14].

Meanwhile, studies on obesity suppression using prebiotics along with probiotics have been conducted [15]. Prebiotics are indigestible, fermentable raw materials that can promote the proliferation of beneficial gut bacteria, or they are “A substrate that is selectively utilized by host microorganisms conferring a health benefit”, as suggested by the International Scientific Association for Probiotics and Prebiotics [16]. Nondigestible polysaccharides, such as inulin, galacto-oligosaccharides (GOS), fructo-oligosaccharides (FOS), lactulose, and resistant starch (RS), are recognized as prebiotics [17]. Prebiotics suppresses the proliferation of harmful bacteria and encourages the growth of healthful bacteria, such as Lactobacilli, to promote the production of short-chain fatty acids (SCFAs) such as acetate, butyrate, and propionate [18]. Among others, resistant starch is one of the fractions thoroughly or partly fermented in the large intestine but cannot be digested in the small intestine of robust people. Suppressing the glycemic response, working as healthy probiotics, lowering cholesterol levels, and boosting the generation of SCFAs in the large intestine is reported as a function of resistant starch [19]. Sweet potato is a good and appropriate resource for the supplement of resistant starch [20]. It was demonstrated that the anti-obesity effects of the gut microbiome are related to lactic acid bacteria [21], and the dietary fiber of sweet potato helps the gut microflora profile in a healthy way [22]. Indeed, the possible correlation between probiotics and obesity has been studied by several researchers, suggesting that a particular phylum or probiotic species can regulate energy metabolism [23]. Lactobacillus is known to induce the degradation (fermentation) of indigestible complex polysaccharides and to promote the efficiency of metabolism in our body. In previous studies, models based on these features proved that these two LAB have remarkable anti-obesity effects [24]. Among LAB, *Lactobacillus rhamnosus* is a strain of Lactobacillus species that are regarded as GRAS strains (generally regarded as safe). In this context, it would be quite probable to obtain a synergistic outcome with enhanced biological activities, such as anti-obesity activity, of Lactobacillus probiotics by using sweet potato as a source of prebiotics simultaneously [25].

To address this hypothesis and to develop a new or better probiotic and/or probiotic-prebiotic combined composition for healthy functional food ingredient or a remedial agent to treat or prevent obesity, The purpose of this study is to compare and make an evaluation of the potential anti-obesity effects of a newly isolated probiotic strain, *Lactobacillus rhamnosus* BST-L.601 (deposited in KCTC under accession number KCTC13517BP), and the fermented product (named SPY) of mashed sweet potato paste (MSPP) with this strain, using 3T3-L1 preadipocyte cells and a high-fat diet (HD)-induced obese C57BL/6 mouse model.

## 2. Materials and Methods

### 2.1. Materials

Dulbecco’s modified Eagle’s medium (DMEM), fetal bovine serum (FBS), and Dulbecco’s phosphate-buffered saline (DPBS) were purchased from Welgene (Gyeongsan, Republic of Korea). Trypsin-ethylenediaminetetraacetic acid (EDTA) and penicillin and streptomycin were purchased from Gibco-BRL (Grand Island, NY, USA). Isobutylmethylxanthine (IBMX), dexamethasone, insulin, Oil red O, and dimethyl sulfoxide (DMSO) were purchased from Sigma-Aldrich (St. Louis, MO, USA). The murine 3T3-L1 preadipocyte cell line (ATCC^®^ CL-173™) was provided by the American Type Culture Collection (Manassas, VA, USA). 3-(4,5-Dimethylthiazolyl)-diphenyl tetrazoliumbromide (MTT) was obtained from DUCHEPA Biochemie (Haarlem, The Netherlands). Antibodies specific to β-actin, CCAAT/enhancer binding protein-α (C/EBPα), peroxisome proliferator-activated receptor-γ (PPAR-γ), adipocyte protein 2 (aP2), fatty acid synthase (FAS), acetyl-CoA carboxylase (ACC) and anti-rabbit IgG-HRP were purchased from Cell Signaling Technology (Danvers, MA, USA). Primers specific to β-actin, PPAR-γ, aP2, FAS, and ACC were purchased from Cosmo Genetech (Seoul, Republic of Korea).

### 2.2. Preparation of Lactobacillus rhamnosus BST-L.601 and Mashed Sweet Potato Medium

*Lactobacillus rhamnosus* BST-L.601 was isolated and identified from the stool of 20 randomly selected Korean people. Human stools were dissolved in germ-free PBS (pH 7.4) with a decimal method, inoculated into De Man, Rogosa, and Sharpe (MRS) agar broth (Becton-Dickinson, Franklin Lakes, NJ, USA), and cultured at 37 °C under anaerobic conditions for 24 to 48 h. After cultivation, the largest colonies were selected, inoculated into MRS broth medium, and cultured at 37 °C under anaerobic and static conditions for 24 to 48 h. From the culture broth showing confluent growth with over 1.0 absorbance at 600 nm, strains were selected and inoculated into skim milk medium (Becton-Dickinson, Franklin Lakes, NJ, USA). The strains showing smooth curd formation were selected and smeared again onto the skim milk agar medium. Finally, a single strain was isolated from a single largest colony. This strain was identified as a strain of *L. rhamnosus* by analyzing the 16S rRNA sequence and deposited in KCTC under accession number KCTC 13517 BP (Korea Patent No. 10-2020-0012236).

A fermented sample (SPY) was prepared by fermentation of mashed sweet potato paste (MSPP) with *L. rhamnosus* BST-L.601. Sweet potato paste was prepared using domestic sweet potato cultivated and collected in Kangwon Province, Republic of Korea. After washing the sweet potatoes, the skin of the sweet potatoes was removed, cut randomly into smaller chips, and finely ground with equal amounts (by weight) of water using a grinder (HR3752/00, Philips, The Netherlands) to make the mashed sweet potato paste (MSPP). Yeast extract (20 g/L, Becton-Dickinson, Franklin Lakes, NJ, USA) was blended with MSPP, and the mixture was finely ground at 10,000 rpm for 10 min using a homogenizer (Daihan Scientific, Wonju, Republic of Korea). The pH of MSPP was adjusted from 6.5 to 8.0 using 1 M HCl before sterilization (15 min, 121 °C), and 40 g/L glucose solution was fortified to the paste after sterilization. *L. rhamnosus* BST-L.601 (1 × 10^6^) CFU was inoculated into sweet potato paste with 40 g/L glucose solution to 10% (*v*/*v*) and fermented at 37 °C for 3 days. Additional fermentation was performed at 4 °C for 3 additional days after the first fermentation.

### 2.3. Cell Culture and Differentiation of Pre-Adipocytes

Murine 3T3-L1 preadipocyte cells (ATCC^®^ CL173) were cultured in preadipocyte medium [PM; a mixture of Dulbecco’s modified Eagle medium (DMEM) added with 10% fetal bovine serum (FBS)] and a 1% penicillin–streptomycin mixture at 37 °C in a humidified atmosphere of 5% CO_2_ until reaching approximately 90% confluence. Unless stated otherwise, preadipocytes were seeded onto 6-well plates at a density of 24,000 cells/well and cultured until 80% confluence was reached. The differentiation of 3T3-L1 preadipocyte cells into mature adipocytes was achieved by culturing cells in differentiation media I [DMEM, 10% FBS, 1 mM dexamethasone, 0.5 mM 3-isobutyl-1-methylxanthine (IBMX), and 10 μg/mL insulin] and differentiation media II [DMEM, 10% fetal bovine serum (FBS), and 10 μg/mL insulin] for 48 h. After that, adipocytes were cultured in DMEM with 10% FBS for original growth and subcultured every 48 h until use.

### 2.4. Cell Viability Determination

Whether L. rhamnosus BST-L.601 and SPY are toxic to 3T3-L1 preadipocyte cells and differentiated mature adipocyte cells was assessed by measuring cell viability using an MTT assay. Cells were exposed to increasing concentrations of each sample (L.601 or SPY). 3T3-L1 preadipocytes (4000 cells/well) were cultured in 96-well plates with SPY at various concentrations (0.05, 0.1, 0.25, and 0.5 mg/mL) dissolved in PM culture medium at 37 °C in a humidified atmosphere of 5% CO_2_ for 48 h. Cell viability was determined by the addition of 50 mL MTT solution (1 mg/mL in phosphate-buffered saline; PBS) to each well and incubation at 37 °C for 4 h. After the culture medium is removed, DMSO was added to each well and incubated at room temperature for 30 min. Absorbance was measured at 570 nm on a microplate reader (Molecular Devices, Seoul, Republic of Korea).

### 2.5. Oil Red O Staining

To examine the effect of SPY on differentiation and lipogenesis, cells were cultured in MDI differentiation medium in 6-well plates, treated with SPY for 8 days, and stained with Oil red O dye. After incubation, cells were washed gently with PBS, fixed with 4% paraformaldehyde for 30 min at room temperature, rinsed with PBS, and then stained with freshly prepared 0.5% (*w*/*v*) Oil red O solution at 37 °C for 1 h. The stained cells were photographed using an inverted microscope (X100, Olympus, Tokyo, Japan) to visualize lipid droplets. To determine the lipid content, the retained dye in adipocytes was extracted with 100% isopropanol and quantified at 517 nm using a microplate reader. Compared to the control, the relative lipid content of each sample was expressed.

The intracellular TG contents were verified by a Cayman Chemical Triglyceride Assay kit (Ann Arbor, MI, USA), as a method of the manufacturer’s instructions. Differentiated adipocytes (Day 8) were treated with increasing amounts of SPY (0.05, 0.1, 0.25, and 0.5 mg/mL) in 6-well plates. The cells were washed and scraped with 200 mL of PBS, and homogenized by sonication for 2 min. Total TG in cell lysates was assayed after that.

### 2.6. Total RNA Preparation and Reverse Transcription-Polymerase Chain Reaction (RT–PCR)

To determine the mRNA expression levels of inducible FAS, ACC, PPAR-γ, and aP2, total RNA from SPY-treated cells was prepared using a total RNA extraction kit (Intron Biotechnology, Republic Korea). RT-PCR was performed using the One-step RT–PCR PreMix kit (Intron Biotechnology, Seongnam-si, Republic of Korea) with appropriate sense and antisense primers for FAS (sense 5′-CGGCTGCAGGTGGTCGATAGG-3′ and antisense 5′-TGTAGGGGTTGCCGCAATGTC-3′), PPAR-γ (sense 5′-GTCTGTGGGGATAAAGCATC-3′ and antisense 5′- CTGATGGCATTGTGAGACAT-3′), ACC (sense 5′-GAAGAGAACAAAAGCGACATG-3′ and antisense 5′-AATGGCTGATAGGAAGATAGA-3′), and β-actin (sense 5’-AGG+TATCCTGACCCTGAAGTACC-3’ and antisense 5’-GTTGCCAATAGTGATGACCTGGC-3’). Primers were amplified under incubation conditions of 95 °C predenaturation for 5 min and 30 cycles of 95 °C denaturation for 30 s, 58 °C annealing for 30 s, 72 °C extension for 40 s, and a final elongation step for 10 min at 72 °C. The products obtained by RT-PCR were separated on a 1.5% agarose gel and stained with ethidium bromide. The gels were then viewed under UV transillumination, and the relative levels of mRNA against β-actin were quantified using ImageJ software from NIH (Bethesda, MD, USA).

### 2.7. Western Blot Analysis

To determine the expression level of adipogenic-related proteins, western blot analysis was performed using the lysates from 3T3-L1 adipocytes cultured in a differentiation medium within or without SPY for 8 days. On Day 8, PBS buffer was used to wash the cells. Cold lysis buffer (pH 7.4) containing 20 mM Tris-HCl, 150 mM NaCl, 1 mM Na2EDTA, 1 mM EGTA, 1% NP-40, 1% sodium deoxycholate, 2.5 mM sodium pyrophosphate, 1 mM β-glycerophosphate, 1 mM Na_3_VO_4_, and 1 mg/mL leupeptin was used to resuspend the cells. The cell lysates were centrifuged at 17,700× *g* and 4 °C for 10 min. The Bradford method (Bio-Rad, Hercules, GA, USA) was applied to determine the protein concentrations. An equal quantity of protein was divided on a 10% SDS-polyacrylamide gel and transferred to PVDF membranes (Bio-Rad, USA). Tris-buffered saline (TBS, pH 7.4) containing 5% nonfat dry milk was used to block the membrane, and primary anti-mouse FAS, aP2, PPAR-γ, and ACC antibodies were incubated with the blocked membrane overnight at 4 °C. The membranes were incubated with anti-mouse IgG-HRP (Cell Signaling Technology) for 2 h at room temperature after washing blocked membranes with TBS containing 0.1% Tween 20. Protein bands were visualized through the ECL system (ABclon, Seoul, Republic of Korea). The ImageJ Program (National Institute of Health, Bethesda, MD, USA) was utilized to quantify the band intensities and to normalize the levels of PPAR-γ, aP2, FAS, and ACC compared to β-actin.

### 2.8. Animal Care and Diets

The Catholic University of Korea approved the animal protocols followed in the present research (Approval Number: 2019-019). Male C57BL/6 mice purchased from Orient Bio Inc. (Gyeonggi-do, Republic of Korea) were kept under controlled temperatures (22–23 °C) on a 12/12-h light-dark cycle. After the acclimatization period, the male C57BL/6 mice were divided into seven groups (n = 8 in each group) and given a normal diet (ND) or high-fat diet (HD) with the indicated doses of samples for 12 weeks, normal diet control group (2018C, Envigo, Indianapolis, IN, USA, 18% calories from fat); HD, high-fat diet (TD.06414, Envigo, USA, 60.5% calories from fat); GAR, HD + 1% *Garcinia cambogia* extract (100 mg/kg); SPY-L, HD + SPY (30 mg, 4 × 10^6^ CFU/kg); SPY-H, HD + SPY (300 mg, 4 × 10^7^ CFU/kg); BST-L.601-L, HD + BST-L.601 (4 × 10^8^ CFU/kg); BST-L.601-H, HD + BST-L.601 (4 × 10^9^ CFU/kg). Each dose of the sample was administered. The *Garcinia cambogia* extract was used as a positive reference. Body weights were measured every week. After 12 weeks of administration of SPY or L.601, the mice were fasted for 24 h and anesthetized with a mixture of alfaxalone (0.15 mL/25 g/mice) and xylazine hydrochloride (0.01 mL/25 g/mice) before sacrifice. Blood serum, adipose tissues, and liver were individually taken for further analyses.

### 2.9. Serum Biochemical Analysis

To collect blood samples, mice were fasted. Blood serum was collected by cardiac puncture and stored at −80 °C before use. To determine levels of total cholesterol (Total-c), high-density lipoprotein cholesterol (HDL-c), and low-density lipoprotein cholesterol (LDL-c) in collected blood serum, LH-1500 Automatic Analyzer (LH-1500, Incheon, Republic of Korea) was utilized.

### 2.10. Chemical Composition Analysis

The general chemical composition, total carbohydrate, protein, polyphenols, and caffeic acid, in the mashed sweet potato paste (MSPP) powder and SPY (the fermented MSPP with BST-L.601), were determined and compared. Total carbohydrate was measured by the phenol-sulfuric acid method at 490 nm using glucose as a reference [26]. Bradford method was operated to quantify total protein and BSA was used as a standard for Bradford method at 595 nm [27]. Total polyphenols were determined by the Folin-Ciocalteu reagent method using gallic acid as a reference at 750 nm [28]. Each powder sample was blended with distilled water to make a 1.0% (*w*/*v*) solution (10 mg/mL) for 1 h using a nutator mixer (FinePCR, Gunpo-si, Republic of Korea) while homogenizing with a sonicator (Branson, MO, USA) by 1 min sonication and 5 min cooling on ice-cold water. The sonication was repeated 10 times. After sonication, each suspension was diluted with distilled water to an appropriate concentration for analysis. The detection and quantification of the amount of caffeic acid in SPY and MSPP were performed by using an HPLC system equipped with a UV detector (1260 Infinity Ⅱ, Agilent, Santa Clara, CA, USA). Samples were dissolved in methanol, fractionated on an XDB-C18 column (150 × 4.6 mm, 5 μm column, Agilent), and eluted at 1.0 mL/min in gradient mode with a mobile phase composed of water (pH 3.15 by formic acid) and acetonitrile.

### 2.11. Statistical Analysis

The results were shown in the means ± SDs for each treatment group in each experiment. Statistical analysis was carried out by using the Statistical Analysis System software package (SAS Institute, Cary, NC, USA). Significance was determined by a one-way analysis of variance, followed by Dunnett’s range test for multiple comparisons, and data were analyzed using the SAS package program (SAS Institute Inc., Cary, NC, USA). Data were considered to be different at *p* < 0.05.

## 3. Results

### 3.1. Effects of SPY and BST-L.601 on the Viability of 3T3-L1 Pre-Adipocytes

**Figure 1:**
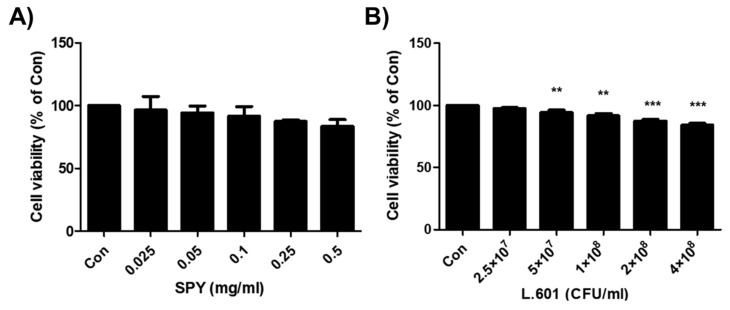
Effects of SPY and BST-L.601 on the viability of 3T3-L1 preadipocyte cells. Cytotoxicity of (**A**) SPY and (**B**) BST-L.601 was assessed by measuring cell viability using the MTT assay after 24 h of exposure to (**A**) SPY (0.025, 0.05, 0.1, 0.25, and 0.5 mg/mL) or (**B**) BST-L.601 (2.5 × 10^7^, 5 × 10^7^, 1 × 10^8^, 2 × 10^8^, and 4 × 10^8^ CFU/mL). The data were expressed as a percentage normalized to sample-untreated control cells (Con). Values are the means ± SDs (n = 8). Values with different superscripts are significantly different among the groups by one-way ANOVA with Dunnett’s multiple comparison test at ** *p* < 0.01; *** *p* < 0.001, compared to the Control group.

To determine whether SPY and BST-L.601 are detrimental to 3T3-L1 preadipocytes, cell survival was evaluated by MTT assay with exposure to increasing doses of SPY (0.025, 0.05, 0.1, 0.25, and 0.5 mg/mL) containing BST-L.601 (2.5 × 10^7^, 5 × 10^7^, 1 × 10^8^, 2 × 10^8^, and 4 × 10^8^ CFU/mL, respectively) (Figure 1A) or BST-L.601 (Figure 1B) for 24 h. As shown in Figure 1, both SPY (Figure 1A) and BST-L.601 (Figure 1B) were not significantly toxic to the growth of 3T3-L1 preadipocytes in the concentration range tested (up to 0.5 mg/mL for SPY and 4 × 10^8^ CFU/mL for BST-L.601).

### 3.2. Effects of SPY on Adipocyte Differentiation

**Figure 2:**
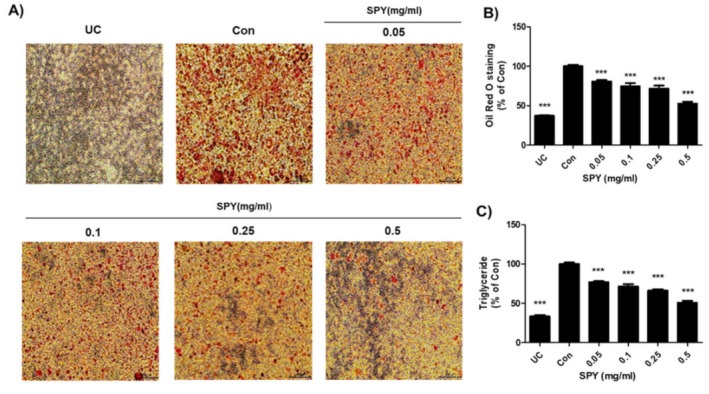
Effects of SPY on lipid accumulation and TG content in differentiating 3T3-L1 cells. Cells were cultured in an MDI differentiation medium and treated with varying concentrations of SPY (0.05, 0.1, 0.25, and 0.5 mg/mL) for 8 days. (**A**) Lipid droplets generated were stained with Oil red O dye and visualized under a microscope (×100). (**B**) Stained lipid droplets were solubilized with isopropanol and quantified at 517 nm on a microplate reader. (**C**) Intracellular triglyceride (TG) contents were measured using a triglyceride assay kit. Data are expressed as the means ± SDs (n = 3). Values with different superscripts are significantly different among the groups by one-way ANOVA with Dunnett’s multiple comparison test at *** *p* < 0.001 compared to the Control group. UC, undifferentiated normal control cells; Con, sample-untreated differentiated control cells.

To assess the efficacy of SPY on inhibiting lipid accumulation, 3T3-L1 preadipocytes were exposed to varying levels of SPY for 8 days, and lipids in adipocytes were stained with Oil red O dye. Figure 2A, the representative images of Oil red O staining, shows that the undifferentiated control cells (UC) were not stained with dye, but huge numbers of stained spots (pink-colored) appeared in the differentiated control cells (Con). This indicates the preadipocytes were differentiated into mature adipocytes, actively synthesizing lipids (Figure 2A). However, these promoted lipids were substantially decreased by SPY (0.05, 0.1, 0.25, and 0.5 mg/mL) in a dose-dependent manner by 19.3%, 25.5%, 28.6%, and 47.4%, respectively, compared to the differentiated control cells (Con, 100%), showing that SPY effectively inhibited the differentiation of adipocyte cells (Figure 2A,B). Moreover, the markedly increased intracellular triglyceride (TG) contents in differentiating adipocytes (Con, 100%) were also effectively reduced to 23.2%, 28.8%, 33.3%, and 50.7% upon SPY (0.05, 0.1, 0.25, and 0.5 mg/mL) exposure (Figure 2C), indicating that SPY inhibited lipid accumulation, especially TG, during adipocyte differentiation.

### 3.3. Inhibitory Effect of SPY on Differentiation and Lipogenesis-Related Protein Expression in 3T3-L1 Cells

**Figure 3:**
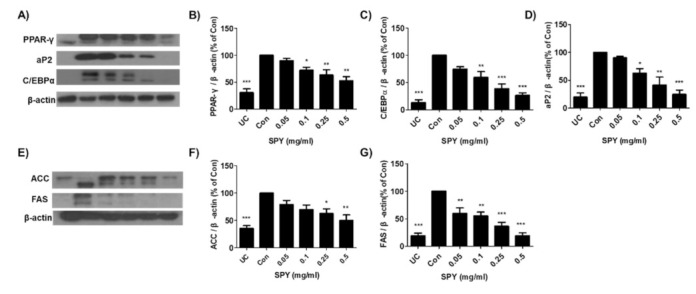
Inhibitory effect of SPY on differentiation and lipogenesis-related protein expression in 3T3-L1 adipocytes. (**A**) and (**E**) 3T3-L1 cells were cultured in MDI differentiation medium for 8 days with increasing concentrations of SPY (0.05, 0.1, 0.25, and 0.5 mg/mL), and cell lysates were used for Western blot analysis. The expression levels of (**B**) PPAR-γ, (**C**) C/EBPα, (**D**) aP2, (**F**) ACC, and (**G**) FAS were quantified by ImageJ software. β-Actin was used as a loading control. Each data point was expressed as the % of control cells (Con, 100%). Data are the means ± SDs (n = 3). Values with different superscripts are significantly different among the groups by one-way ANOVA with Dunnett’s multiple comparison test at * *p* < 0.05; ** *p* < 0.01; *** *p* < 0.001, compared to the Control group. UC, undifferentiated control cells; Con, untreated sample, differentiated control cells.

Throughout adipogenesis, factors and proteins related to adipocytes such as C/EBPα, PPAR-γ, and aP2 are expressed [23]. C/EBPα and PPAR-γ stimulate the expression of lipogenic enzymes similar to FAS and ACC in various ways during adipogenesis [16,24]. To determine the effects of SPY on the expression of these adipogenic and lipogenic marker proteins, 3T3-L1 cells were cultured in an MDI differentiation medium for 8 days with the indicated concentration of SPY. Western blot analysis showed SPY treatment (0.05, 0.1, 0.25, and 0.5 mg/mL) resulted in predominant inhibition in the expression of these proteins (Figure 3). SPY (0.5 mg/mL) considerably inhibited the protein levels of PPAR-γ by 48.4% (Figure 3A,B), C/EBPα by 73.7% (Figure 3A,C), and aP2 by 75.7% (Figure 3A,D) when it is compared with the differentiated, Sample-untreated control cells (Con, 100%). SPY treatment (0.5 mg/mL) also significantly inhibited the expression of ACC by 50.0% (Figure 3E,F) and FAS by 80.9% (Figure 3E,G) compared to the differentiated but SPY-untreated group (Con, 100%). These results proved SPY effectively suppressed the differentiation of preadipocytes through the downregulation of diverse adipogenesis-specific transcription factors and lipogenesis marker proteins.

### 3.4. Body and Organ Weight Changes of Mice Fed the Different Diets

**Figure 4:**
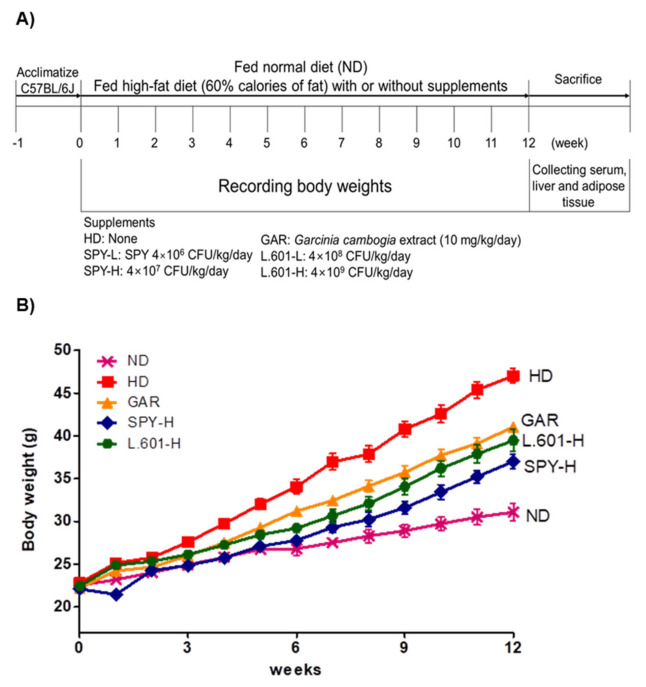
Protocol for animal treatment and body weight change of mice fed the different diets. (**A**) ND, normal diet; HD, high-fat diet; GAR, HD supplemented with *Garcinia cambogia* extract (100 mg/kg); SPY−L, a high-fat diet supplemented with SPY (4 × 10^6^ CFU/kg); SPY− H, HD supplemented with SPY (4 × 10^7^ CFU/kg); L.601− L, HD supplemented with BST-L.601 (4 × 10^8^ CFU/kg); L.601− H, HD supplemented with BST-L.601 (4 × 10^9^ CFU/kg). L.601, *L. rhamnosus* BST-L.601. (**B**) Body weight change of mice fed the different diets. Mice were given their diets and indicated doses of samples for 12 weeks, and the body weight was weighed every week. Values are the means ± SDs (n = 8).

C57BL/6 mice were fed a high-fat diet (HD) for 12 weeks and used as the HD-induced obese model. Mice were randomly divided into 7 groups (n = 8): ND, normal diet group; HD, high-fat diet (control); GAR, HD + 1% *Garcinia cambogia* extract-treated group (100 mg/kg); SPY-L, HD + SPY (4 × 10^6^ CFU/kg); SPY-H, HD + SPY (4 × 10^7^ CFU/kg); L.601-L, HD + BST-L.601 (4 × 10^8^ CFU/kg); L.601-H, HD + BST-L.601 (4 × 10^9^ CFU/kg). Oral administration of diet for mice with or without doses of samples proceeded for 12 weeks, and the body weight of each mouse was checked once a week. As shown in Figure 4B, HD-induced obese mice had an increased body weight by 48.0% after 12 weeks of feeding, and the SPY-H group and L.601-H group had decreased body weight by 19.6% and 14.8%, respectively, compared with the HD group. The relative weights of epididymal fat, visceral fat, abdominal fat, and liver tissue in the HD group were higher than those in the ND group (Table 1). The administration of SPY and BST-L.601 decreased the weight of total fat tissues (epididymal, visceral and subcutaneous, and abdominal fat tissues) by 29.8% (SPY-H) and 25.4% (L.601-H), respectively (Table 1).

### 3.5. SPY Prevents Hyperlipidemia in High-Fat Diet Mice

**Figure 5:**
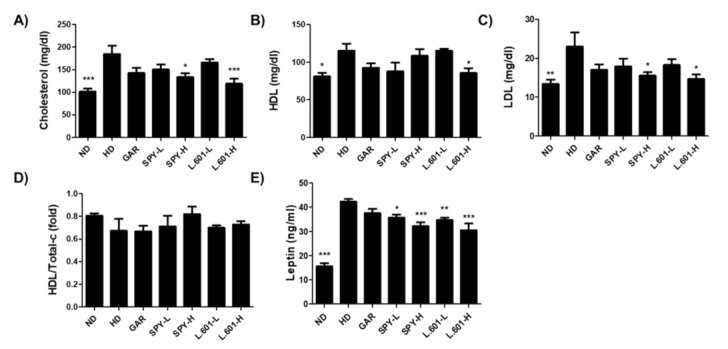
Effects of SPY and BST-L.601 on hyperlipidemia markers. The blood serum of HD-induced obese mice was analyzed by an automated analyzer after oral administration of various diets for 12 weeks. (**A**) Total cholesterol (Total-c), (**B**) high-density lipoprotein-cholesterol (HDL), (**C**) low-density lipoprotein-cholesterol (LDL), (**D**) HDL/Total-c, (**E**) Leptin. ND, normal diet; HD, high-fat diet; GAR, HD supplemented with *Garcinia cambogia* extract (10 mg/kg); SPY−L, HD supplemented with SPY (4 × 10^6^ CFU/kg); SPY− H, HD supplemented with SPY (4 × 10^7^ CFU/kg); L.601− L, HD supplemented with BST-L.601 (4 × 10^8^ CFU/kg); L.601− H, HD supplemented with BST-L.601 (4 × 10^9^ CFU/kg). L.601, *L. rhamnosus* BST-L.601. Values are the means ± SDs (n = 8). Values with different superscripts are significantly different among the groups by one-way ANOVA with Dunnett’s multiple comparison test at * *p* < 0.05; ** *p* < 0.01; *** *p* < 0.001, compared to the HD group.

To assess the effects of SPY and BST-L.601 on the serum biochemical parameters in HD-induced obese mice, total cholesterol (Total-c), high-density lipoprotein cholesterol (HDL), low-density lipoprotein cholesterol (LDL), and leptin were analyzed by automated analyzer after various diets were orally administered to HD-induced obese mice. SPY (SPY-H) reduced the cholesterol and LDL levels by 27.9% and 32.6%, respectively, and BST-L.601 (L.601-H) reduced cholesterol and LDL levels by 35.7% and 36.4%, respectively (Figure 5A,C) compared with the HD group (100%). Oral administration of SPY and BST-L.601 (L.601-H) increased the ratio of HDL/Total-c level by 21.9% and 8.3%, respectively (Figure 5D). SPY also decreased the secretion of leptin, one of the hormones secreted from visceral and subcutaneous adipose tissue. Leptin secretion was decreased in the SPY-H group by 24.0% and in the L.601-H group by 27.8%, respectively (Figure 5E), when the secretion of leptin in the HD group is set at 100%. These results indicated that SPY and BST-L.601 effectively regulate cholesterol levels in blood serum.

### 3.6. Effects of SPY and BST-L.601 on the Size and Numbers of Adipocytes in Liver and Epididymal Fat Tissues

**Figure 6:**
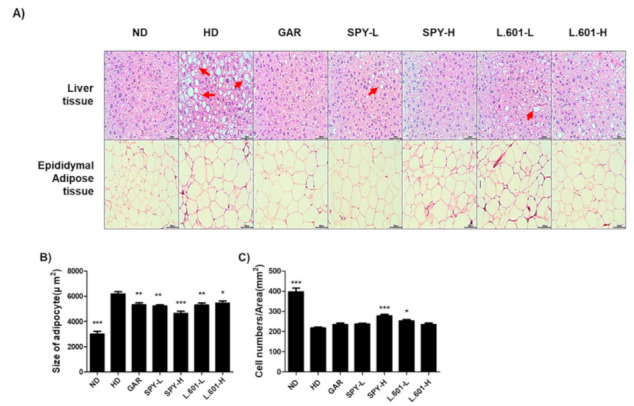
Effect of SPY and BST-L.601 on the size and numbers of adipocytes in liver and epididymal fat tissues in HD-induced obese mice. Liver tissues and adipose tissues were stained with hematoxylin and eosin (H&E). (**A**) Sections of stained epididymal adipose tissue and liver tissue were monitored under a light microscope (magnification, ×200 for epididymal adipose tissues, ×400 for liver tissues). (**B**) Size of epididymal adipose tissue and (**C**) cell numbers in the measured area (measured unit area = 1.48 μm^2^). ND, normal diet; HD, high-fat diet; GAR, HD supplemented with *Garcinia cambogia* extract (100 mg/kg); SPY−L, HD supplemented with SPY (4 × 10^6^ CFU/kg); SPY− H, HD supplemented with SPY (4 × 10^7^ CFU/kg); L.601− L, HD supplemented with BST-L.601 (4 × 10^8^ CFU/kg); L.601− H, HD supplemented with BST-L.601 (4 × 10^9^ CFU/kg). L.601, *L. rhamnosus* BST-L.601. Values with different superscripts are significantly different among the groups by one-way ANOVA with Dunnett’s multiple comparison test at * *p* < 0.05; ** *p* < 0.01; *** *p* < 0.001, compared to the HD group.

The increment of the mass and the number of adipocytes mainly depends on the amount of lipids accumulated in the fat cells. Therefore, the size and number of adipocytes accumulated in epididymal adipose tissue and liver tissue are habitually evaluated indicators to assess the potential anti-obesity efficacy of ingredients [24]. To determine the numbers and sizes of adipocytes in liver tissues and fat tissues, tissues were stained by hematoxylin and eosin staining (H&E). Sections of epididymal adipose tissue of the HD group revealed an increased number of expanded adipocytes (red-arrow) following H&E staining is completed (Figure 6A). However, administration of SPY (SPY-H) and BST-L.601 (L.601-H) significantly reduced these enlarged cell sizes of adipocytes by 25.0% and 11.0%, respectively, and the number of adipocytes per 1.48 μm^2^ was increased by 28.5% and 7.6%, respectively (Figure 6B,C).

### 3.7. SPY and BST-L.601 Suppress Adipogenic and Lipogenic Marker Protein mRNA Expression in HD-Induced Obese Mice

**Figure 7:**
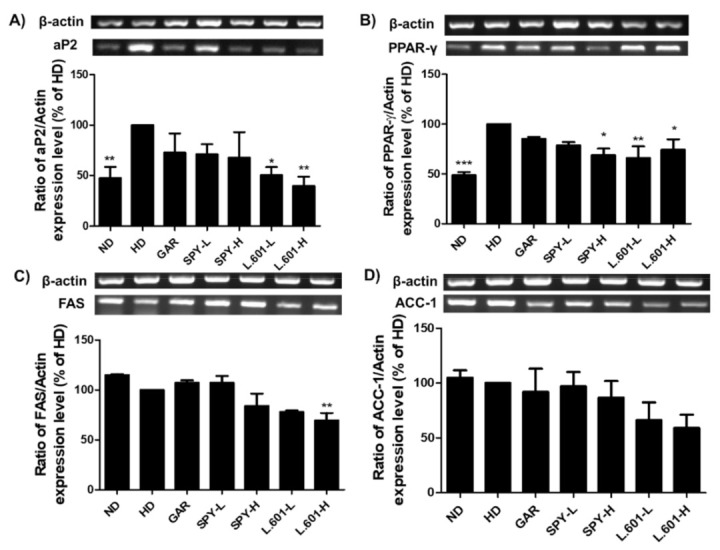
Effects of SPY and BST-L.601 on the mRNA levels of lipogenic markers in the liver tissue of HD-induced obese mice. To investigate the effects of SPY and BST-L.601 on the expression of (**A**) and (**B**) adipogenic markers (aP2 and PPAR-γ) and (**C**) and (**D**) lipogenic marker proteins (FAS and ACC-1), liver tissues of HD-induced obese mice treated with various diets for 12 weeks were homogenized and processed for RT-PCR and quantified by ImageJ software. The relative mRNA levels of lipogenic markers in the liver tissue were measured by RT-PCR. ND, normal diet; HD, high-fat diet; GAR, HD supplemented with *Garcinia cambogia* extract (100 mg/kg); SPY–L, HD diet supplemented with SPY (4 × 10^6^ CFU/kg); SPY–H, HD supplemented with SPY (4 × 10^7^ CFU/kg); L.601–L, HD supplemented with BST-L.601 (4 × 10^8^ CFU/kg); L.601–H, HD supplemented with BST-L.601 (4 × 10^9^ CFU/kg). L.601, *L. rhamnosus* BST-L.601. Values with different superscripts are significantly different among the groups by one-way ANOVA with Dunnett’s multiple comparison test at * *p* < 0.05; ** *p* < 0.01, *** *p* < 0.001, compared to the HD group.

RT-PCR analysis was performed to identify the effects of SPY and BST-L.601 on the expression levels of adipogenic markers in HD-induced obese mice. After 12 weeks of oral administration of SPY and BST-L.601, liver tissues were lysed, and the amount of mRNA was measured. Oral administration of SPY (SPY-H) and BST-L.601 (L.601-H) decreased the mRNA expression level of aP2 by 32.3% and 60.1% (Figure 7A), PPAR-γ by 31.0% and 25.5% (Figure 7B), FAS by 15.9% and 30.3% (Figure 7C), and ACC-1 by 13.3% and 41.0%, respectively (Figure 7D). These results suggested the exhibition of the anti-obesity ability of SPY and BST-L.601 through the downregulation of the expression of various adipogenesis-specific transcription factors and lipogenesis marker proteins.

### 3.8. SPY and BST-L.601 Suppress Adipocyte Differentiation and Lipogenic Marker Protein Expression in Liver Tissue of HD-Induced Obese Mice

**Figure 8:**
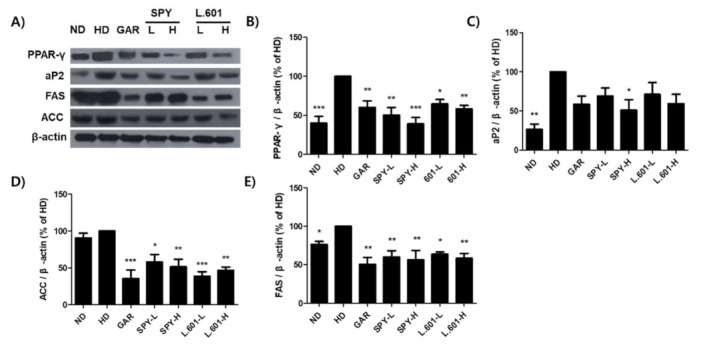
Effects of SPY and BST-L.601 on adipocyte differentiation and lipogenic marker proteins in the liver tissue of HD-induced obese mice. (**A**) Western blot analysis and ImageJ software were used to monitor and quantify the expression levels of adipocyte differentiation and lipogenic marker proteins, (**B**) PPAR-γ, (**C**) aP2, (**D**) ACC, and (**E**) FAS. β-Actin was used as a loading control. Each data point was expressed as the % of the HD group (100%). ND, normal diet; HD, high-fat diet; GAR, a high-fat diet supplemented with *Garcinia cambogia* extract (100 mg/kg); SPY−L, HD supplemented with SPY (4 × 10^6^ CFU/kg); SPY− H, HD supplemented with SPY (4 × 10^7^ CFU/kg); L.601− L, HD supplemented with BST-L.601 (4 × 10^8^ CFU/kg); L.601− H, HD supplemented with BST.L-601 (4 × 10^9^ CFU/kg). L.601, *L. rhamnosus* BST-L.601. Values with different superscripts are significantly different among the groups by one-way ANOVA with Dunnett’s multiple comparison test at * *p* < 0.05; ** *p* < 0.01; *** *p* < 0.001, compared to the HD group.

To determine the effect of SPY and BST-L.601 on adipogenic and lipogenic marker proteins in HD-induced obese mice, the levels of adipocyte differentiation transcription factors as PPAR-γ and aP2 and lipogenic enzymes similar to ACC and FAS were investigated by Western blotting. SPY (SPY-H group) and BST-L.601 (L.601-H group) were shown to reduce PPAR-γ by 60.8% and 41.9% (Figure 8A,B) and aP2 by 48.8% and 40.7% (Figure 8A,C) when the β-actin level in the HD group was set at 100%. Administration of SPY (SPY-H) and BST-L.601 (L.601-H) also decreased ACC by 48.5% and 53.5% (Figure 8A,D) and FAS by 43.8% and 41.7% (Figure 8A,E). These results showed that SPY and BST-L.601 effectively downregulated adipogenesis and lipogenesis.

### 3.9. Chemical Composition Profile of SPY and MSPP

The chemical composition of MSPP and SPY was determined as summarized in Table 2. The MSPP and SPY sample preparations contained total carbohydrates up to 922.4 mg/g (92.24%, *w*/*w*) and 810.5 mg/g (81.05%), protein up to 30.7 mg/g (0.0307%) and 16.3 mg/g (0.0163%), and total polyphenols up to 1.65 mg/g (0.00165%) and 1.99 mg/g (0.00199%), respectively, indicating that both samples consist mostly of carbohydrates (Table 2). One of the possible explanations for the decreased level of total carbohydrates in SPY, compared to that of MSPP, would be that some of the digestible carbohydrates were consumed for the growth and proliferation of BST-L.601 during fermentation.

**Figure 9:**
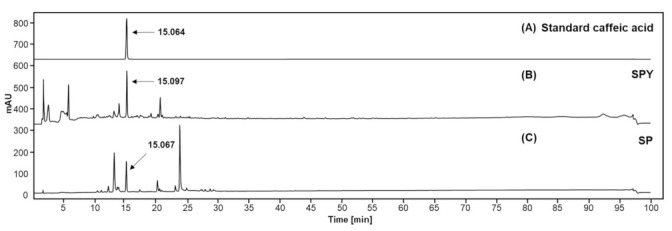
HPCL analysis for caffeic acid in SPY and MSPP. (**A**) Authentic caffeic acid (Sigma, San Francisco, CA, USA). (**B**) SPY and (**C**) SP (MSPP) samples dissolved in methanol were separately fractionated on an XDB-C18 column and detected by a UV detector at 330 nm and quantified by comparing the peak area on the chromatogram set to the peak area of standard caffeic acid at 100% as described in the manuscript.

The results from HPLC analysis for caffeic acid in MSPP (SP) and SPY confirmed the presence of caffeic acid in both samples up to 0.26 mg/g (MSPP) and 0.18 mg/g (SPY), respectively (Figure 9 and Table 2). One of the bioactive polyphenolic compounds of plant origin, caffeic acid (CFA) has been known to exert effects in anti-obesity by reducing body weight and regulating gut microbiota [29]. Therefore, in addition to BST-L.601 in SPY, CFA might contribute to the anti-obesity effects of SPY shown in this study, at least partly. Additionally, the presence of CFA in sweet potatoes was reported [30,31,32]. This suggests that CFA present in SPY can be utilized as an index compound for SPY and SPY-based products.

## 4. Discussion

Several researchers have reported that oral medication of lactic acid bacteria (LAB) with prebiotics promotes the intestinal activity of LAB [22]. It was reported that the anti-obesity efficacy of the gut microbiome is related to LAB, and the dietary fiber from sweet potatoes helps a gut microbiome profile in a healthy way [21,22]. In this study, a newly isolated probiotic strain, *Lactobacillus rhamnosus* BST-L.601 (KCTC13517BP), and its fermented product (named SPY) with mashed sweet potato paste (MSPP) were evaluated to prove the efficacy of anti-obesity through using 3T3-L1 preadipocyte cells and an HD-induced obese mouse model.

Obesity is caused by the accumulation of triglycerides in adipocytes during the differentiation of 3T3-L1 preadipocytes [30]. Previous studies have shown that the first factors expressed after MDI treatment are C/EBPβ and C/EBPγ [33]. These two factors promote the expression of C/EBPα and PPAR-γ in the middle and end of preadipocyte differentiation [34]. Among them, C/EBPα is an essential factor inducing the inhibition of lipogenesis and adipogenesis. In addition, the decrease in the expression levels of C/EBPα and PPAR-γ induces a decrease in the expression of adipose protein 2 (aP2), which is known as a differentiation factor of preadipocytes, and PPAR-γ is a major factor involved in lipogenesis and adipogenesis [33]. The decrease in the expression level of these two factors leads to proteins that transport fatty acids to cells, causing lipid generation in cells [35]. This research showed that treatment of SPY decreased lipid droplets in 3T3-L1 preadipocytes and it means that SPY suppresses the adipogenesis of 3T3-L1 preadipocytes. Moreover, the expression of C/EBPα and PPAR-γ, which are the major adipogenesis factors, was significantly suppressed by SPY. Therefore, these results imply that SPY could negatively control the adipogenesis of 3T3-L1 preadipocytes.

Acetyl-CoA carboxylase (ACC) inhibits the activity of AMP-activated protein kinase (AMPK), reducing the production of malonyl-CoA [36,37]. Malonyl-CoA is synthesized as palmitate by fatty acid synthase (FAS), and palmitate is known to reduce fat accumulation and inhibit lipolysis [38,39]. In this research, oral administration of SPY reduced the expression of ACC and FAS, demonstrating its ability to downregulate lipogenesis. These results mean that SPY controls the expression of ACC and FAS, and also can be an effective agent to control adipogenic and lipogenic metabolism in 3T3-L1 preadipocytes. In previous research, it was proven that one of the *L. rhamnosus* strains controls the expression of transcription factors associated with adipogenic metabolism in an HD-induced obese mouse model [40].

The observed in vitro anti-obesity potential of SPY was further confirmed *in* an in vivo model system using HD-induced obese mice. The results showed that the significantly elevated body weight in HD mice was effectively reduced by oral administration of SPY or BST-L.601, with a more significant reduction by SPY than BST-L.601 treatment. The SPY used in this study was prepared by fermentation of mashed sweet potato paste (MSPP) with the BST-L.601 strain. Similar to our present observation, a combined preparation of prebiotics and probiotics was reported to reduce body fats [41]. LAB is demonstrated that it has efficacy in reducing hypercholesterolemia and preventing obesity [42]. Previously, it was reported that sweet potatoes contain significant amounts of resistant starch, caffeic acid, and many other compounds, including phenolic compounds, anthocyanins, and caffeoyl compounds [30,31,32]. One of the bioactive polyphenolic compounds of plant origin, caffeic acid (CFA) has been known to have various pharmacological activities such as anti-inflammatory, anti-cancer, anti-oxidant, and anti-obesity effects. Anti-obesity effectiveness of CFA is mediated by reducing body weight and regulating the gut microbiome in obese mice [29]. Resistant starch is known to express some beneficial effects related to metabolic syndrome such as inhibiting the increment of blood cholesterol and decreasing the glycemic response. Moreover, resistant starch acts as a functional prebiotic [19]. Therefore, although it is not proven right now, the observed results that SPY-H was more effective in decreasing the body weight gain in HD mice than treatment with BST-L.601 alone would be due to the presence of certain compounds contained in MSPP, such as resistant starch and CFA, which could synergistically contribute to the lowering of body weight gain in HD mice. Alternatively, it would also be possible that SPY may contain some other compounds produced during the fermentation of MSPP with BST-L.601 probiotics. On the other hand, hydroxycitric acid (HCA) is one of the key bioactive chemicals in *Garcinia cambogia*, and its anti-obesity effect has been explained unclearly for the last decades; thus, *Garcinia cambogia* has been widely added as a main raw material for anti-obesity functional foods [43]. In a previous study performed by another research group, it was confirmed that the administration of *Garcinia cambogia* powder (1%, *w*/*w*, 60% HCA) had an effect on inhibiting fat accumulation without toxicity [44,45]. For the above reasons, *Garcinia cambogia* was chosen as a positive control of anti-obesity efficacy for comparison with SPY. The results showed that SPY also exerted a significant positive effect on weight loss in fatty liver, epididymal adipose tissue, and visceral fat, which was comparable to that of the GAR (*Garcinia cambogia* extract, 100 mg/kg) group.

Meanwhile, Oil red O staining and TG content measurement in differentiating adipocyte cells showed that SPY significantly decreased lipid accumulation. Furthermore, histopathological analysis of adipose tissue located on the liver and epididymis in HD-induced obese mice by H&E staining demonstrated that administration of SPY-H and BST-L.601-H significantly reduced the enlarged cell size and increased the cell numbers of adipocytes in these tissues. One of the indicators for anti-obesity assessment is the size and number of adipocytes. Especially, adipocytes accumulated in epididymal and liver tissue are a useful index for verifying anti-obesity efficacy from chemicals or natural ingredients [46]. The important factor to define obesity is an increment of adipose tissue mass driven by either an explosive increment of the number or size of adipocytes [4]. Hypertrophy is mainly due to the accumulation of lipids and the hyperplasia caused by the proliferation and differentiation of adipocyte precursor cells into mature adipocytes [4]. This process is called adipogenesis. Thus, the regulation of the adipose tissue mass is conducted by suppressing adipogenesis, reducing lipogenesis, and enhancing lipolysis. An additional method to control adipose tissue is inducing apoptotic death of cells [5]. Therefore, the results of our study that SPY and BST-L.601 significantly reduced the enlarged cell size and increased the cell numbers of adipocytes in these tissues suggest that both SPY and BST-L.601 exert anti-obesity effects by effectively reducing adipose tissue mass by inhibiting lipogenesis of adipocytes.

On the other hand, as shown by H&E staining of liver tissues, oral administration of SPY and BST-L.601 prominently declined the immoderate generation and deposit of lipids in hepatocytes. This suggests that these materials may be potentially effective against hepatic steatosis, which is an important factor to define nonalcoholic fatty liver disease (NAFLD) [47]. Several animal studies using rodents have demonstrated that suppressing the accumulation of lipids in the liver tissue could be for hepatoprotection from HD-induced NAFLD [48,49]. The results of this study imply that SPY is useful for weight loss in fatty liver, epididymal adipose tissue, and visceral fat in HD-induced obese mice. Although it needs to be clarified through the following research, SPY, and BST-L.601 may exert protective effects to inhibit hepatic steatosis and thus NAFLD in HD-fed mice, which would be an additional health benefit of SPY and BST-L.601.

SPY was also shown to suppress the secretion of leptin, an adipocyte hormone that is secreted by adipocytes in response to their triglyceride levels, and is known to regulate energy expenditure and food intake [50,51]. Circulating leptin levels in the blood are associated with the extent of obesity; thus, leptin is a sensitive biomarker to indicate obesity [51,52]. In this study, oral administration of SPY and BST-L.601 to HD-induced obese mice for 12 weeks resulted in reduced levels of leptin in blood serum, suggesting that both materials, SPY, and BST-L.601, attenuated the secretion of leptin, thereby downregulating lipid metabolism in HD-fed mice.

## 5. Conclusions

This study revealed that, with no detectable level of cytotoxicity, SPY, a fermented product (named SPY) of *Lactobacillus rhamnosus* BST-L.601 in mashed sweet potato paste (MSPP) medium, significantly reduced the lipid accumulation and TG contents in 3T3-L1 adipocytes. Moreover, SPY also inhibited the differentiation of adipocytes and lipogenesis through suppression of the expression of adipogenesis-related markers such as C/EBPα, PPAR-γ, and aP2 and fatty acid synthetic pathway proteins such as ACC and FAS. In HD-induced obese mice, oral administration of SPY or BST-L.601 for 12 weeks significantly reduced the body and liver weight, the size of adipocytes, and the weight of epididymal, visceral, and subcutaneous fat tissues. Administration of SPY or BST-L.601 also reduced the serum levels of total cholesterol and LDL cholesterol and leptin secretion. These results indicated that both SPY and BST-L.601 effectively suppress HD-induced adipogenesis and lipogenesis through the downregulation of the expression of adipogenic marker proteins and lipogenesis-related marker proteins. SPY was more effective in decreasing body weight gain in HD mice than in treatment with BST-L.601 alone. The results of the present study suggest that SPY and BST-L.601 can be potential candidates as active ingredients to develop health-beneficial functional foods or new probiotic-prebiotic combined compositions to prevent or treat obesity.

## Figures and Tables

**Table 1:** Changes in organ and fat tissue weights.

Group	ND	HD	GAR	SPY−	SPY−	L.601−	L.601−
Liver weight (g)	1.59 ± 0.17	1.93 ± 0.44	1.61 ± 0.16	1.71 ± 0.47	1.49 ± 0.19 *	1.52 ± 0.19 *	1.49 ± 0.17 *
Kidney weight (g)	0.45 ± 0.06	0.45 ± 0.04	0.48 ± 0.04	0.47 ± 0.08	0.49 ± 0.06	0.43 ± 0.06	0.48 ± 0.04
Spleen weight (g)	0.081 ± 0.016	0.083 ± 0.007	0.079 ± 0.012	0.078 ± 0.009	0.079 ± 0.007	0.072 ± 0.01	0.074 ± 0.012
Epididymis fat tissue (g)	1.029 ± 0.26 ***	3.246 ± 0.55	3.151 ± 0.21	2.920 ± 0.24	2.725 ± 0.41	3.446 ± 0.14	2.533 ± 0.48 **
Visceral fat tissue (g)	0.449 ± 0.11 ***	1.752 ± 0.17	1.394 ± 0.19 **	1.480 ± 0.17 *	1.321 ± 0.12 ***	1.578 ± 0.33	1.240 ± 0.11 ***
Abdominal fat tissue (g)	0.889 ± 0.19 ***	4.797 ± 0.37	3.512 ± 0.56 ***	3.855 ± 0.39 **	3.035 ± 0.56 ***	3.849 ± 0.34 **	3.373 ± 0.85 ***
Total fat (g)	2.48 ± 0.66 ***	10.14 ± 1.06	7.92 ± 0.67 **	8.35 ± 0.62 *	7.12 ± 1.11 ***	8.50 ± 0.46	7.57 ± 1.93 ***

ND, normal diet; HD, high-fat diet; GAR, HD supplemented with *Garcinia cambogia* extract (10 mg/kg); SPY–LHD supplemented with SPY (4 × 10^6^ CFU/kg); SPY–H, HD supplemented with SPY (4 × 10^7^ CFU/kg); L.601–L, HD supplemented with BST-L.601 (4 × 10^8^ CFU/kg); L.601–H, HD supplemented with BST-L.601 (4 × 10^9^ CFU/kg). L.601, *L. rhamnosus* BST.-L.601. Values are the means ± SDs (n = 8). Values with different superscripts are significantly different among the groups by one-way ANOVA with Dunnett’s multiple comparison test at * *p* < 0.05; ** *p* < 0.01; *** *p* < 0.001, compared to the HD group.

**Table 2:** Chemical composition analysis.

	Total Carbohydrate ^A^ (mg GE/g)	Total Protein ^B^ (mg BE/g)	Total Polyphenols ^C^ (mg GAE/g)	Caffeic Acid (mg/mL)
MSPP ^D^	922.4 (±7.69)	30.7 (±1.69)	1.65 (±0.01)	0.26
SPY	810.5 (±17.40)	16.3 (±1.00)	1.99 (±0.01)	0.18

^A^ Total carbohydrate was measured by the phenol-sulfuric acid method and expressed as glucose equivalent (GE) in 1 g of dry sample. ^B^ Total protein was quantified by the Bradford method and expressed as BSA equivalent (BE) in 1 g of dry sample. ^C^ Total polyphenols were determined by the Folin-Ciocalteu reagent method and expressed as gallic acid equivalents (GAE) in 1 g of dry sample. ^D^ MSPP, mashed sweet potato paste. SPY, the fermented product of MSPP with *L. rhamnosus* BST-L.601.

## Data Availability

The data presented in this study are available on request from the corresponding author.
